# Knowledge, Attitudes, and Practices Regarding Natural Disaster Management Among Dentists in Southeastern Iran: A Cross‐Sectional Study

**DOI:** 10.1002/hsr2.72923

**Published:** 2026-07-29

**Authors:** Fatemeh Jahanimoghadam, Fatemeh Karami Nadik, Amir Mohammad Sabzi, Parya Jangipour Afshar, Hojjat Farahmandnia

**Affiliations:** ^1^ Social Determinants on Oral Health Research Center Kerman University of Medical Sciences Kerman Iran; ^2^ Physiology Research Center, Institute of Neuropharmacology Kerman University of Medical Sciences Kerman Iran; ^3^ School of Dentistry Shiraz University of Medical Sciences Shiraz Iran; ^4^ HIV/STI Surveillance Research Center, and WHO Collaborating Center for HIV Surveillance, Institute for Futures Studies in Health Kerman University of Medical Sciences Kerman Iran; ^5^ Health in Disasters and Emergencies Research Center, Institute for Futures Studies in Health Kerman University of Medical Sciences Kerman Iran

**Keywords:** attitude, dentists, disaster response, disaster risk management, knowledge, natural disasters, practice

## Abstract

**Background and Aims:**

Disasters pose a significant concern for healthcare systems. Dentists, as essential members of healthcare teams, possess skills and capabilities valuable for disaster risk management. However, little is known about the knowledge, attitudes, and practices (KAP) of natural disaster risk management among dentists in Iran. This study was conducted to evaluate dentists' KAP of natural disaster risk management and to determine the correlation between these variables.

**Methods:**

This cross‐sectional study involved 403 general and specialist dentists from Kerman city in southeastern Iran. We evaluated dentists' KAP of natural disaster risk management using a researcher‐designed questionnaire and convenience sampling.

**Results:**

The results showed that dentists had high knowledge (12.96 ± 1.54, 92.6%), desirable attitudes (46.42 ± 2.45, 99.3%), and desirable practices (17.63 ± 1.89, 94%). Higher knowledge was observed among dentists aged over 46 years, married participants, those working in private and public clinics, and those with more than 21 years of experience. Positive attitudes were more common among married dentists, those employed in both private and public clinics, and those with less than 10 years of work experience. Multivariate analysis further showed that age and working in both private and public clinics were significant predictors of higher knowledge, while employment in both settings was associated with a more positive attitude.

**Conclusion:**

Given the high levels of knowledge, positive attitudes, and effective practices among Iranian dentists in disaster risk management, they can play a pivotal role in emergency response. To utilize this capacity, it is essential to formally recognize and integrate the participation of dentists into the national disaster management system. This integration requires establishing standardized protocols and interdisciplinary training programs to better incorporate the dental profession into Iran's emergency response structure.

**Abbreviations**
KAP: knowledge, attitudes, and practices

## 1 Introduction

Disasters are significant disruptions to a community or society resulting from hazardous events that interact with exposure, vulnerability, and capacity, leading to various losses and impacts on people, materials, the economy, and the environment [1, 2]. Disasters pose significant concerns for health systems, and disaster management‐focused health systems aim to be ready for and handle mass casualty and catastrophic events in their communities [3]. Given the extensive disruptions caused by disasters, the knowledge, attitudes, and practices (KAPs) of natural disaster risk management among all healthcare providers are crucial. Assistance from various healthcare departments is essential for providing predefined, structured, and systematic aid to victims [4]. It is imperative for members of public health systems and the medical community to understand that dental professionals can provide valuable supplementary support during sudden events and disaster management.

Dentists and dental assistants, as part of the healthcare team, can use their competencies, skills, and dedication to help in disaster management [5, 6]. Moreover, a strong KAP of natural disaster risk management enables dentists to contribute effectively by responding to disasters and controlling infections, using medical history to provide care, reading X‐rays, administering injections, suturing wounds, managing infections, prescribing medication, making diagnoses based on signs and symptoms, and prioritizing patients for treatment based on severity [7, 8]. However, the role of dentists has frequently been overlooked in most studies examining the understanding and preparedness of healthcare providers in disaster management, especially natural disasters [9].

The literature review confirms the valuable role of dentists in public health disasters. For example, Galligan [8] noted that dentists could assist by educating patients on symptoms and informing public health authorities about potential exposures. Gambhir et al. (2021), in a review study, reported that dentists have important clinical skills and medical knowledge that are valuable in disaster management, recommending specialized training to increase their effectiveness during emergencies and surge events [10]. Odai et al. [11] in Nigeria revealed a high level of willingness and a positive attitude towards disaster management among dentists. However, dentists had low levels of knowledge, behavior, and preparedness in disaster management. A study in the United Arab Emirates (2023) revealed that healthcare practitioners, including dentists, physicians, and nurses, had moderate knowledge, positive attitudes, and high readiness for medical disaster management [12]. A multicenter study conducted in ten Caribbean countries during the COVID‐19 pandemic reported dentists' awareness of viral spread and their adherence to international guidelines on infection control [13]. A study in the USA (2024) found that targeted education improved dental hygiene and dental assistant students' knowledge, confidence, and attitudes toward disaster victim identification and disaster medicine management [14].

Research highlights the significant, yet underutilized, potential of dentists in disaster management. By leveraging their specialized clinical and forensic competencies, interdisciplinary emergency teams can improve response outcomes, provided that targeted training and awareness initiatives are implemented. Limited studies have focused on dentists' KAP of disaster risk management, and there is a need to investigate these factors based on the type of hazard [15, 16].

To effectively fulfill the role of dentists in disaster risk management, especially during natural disasters, appropriate baseline KAP, physical and psychological preparedness, training, and retraining are essential. Assessing these factors and the readiness of dentists is crucial to demonstrate the need for targeted training among healthcare providers [17, 18]. Currently, the number of available studies that evaluate healthcare dentists' KAP, preparedness, and perspectives on disaster management is limited [19, 20]. However, we could not find any study on Iranian dentists' KAP of natural disaster management or the correlation among these variables.

Given the crucial role of dentists and dental assistants in community disaster management, fundamental information is necessary from different societies. Therefore, the present study aimed to evaluate dentists' KAP of natural disaster management, determine the correlation among these variables, and identify significant predictors of KAP using multivariate linear regression. By gathering fundamental and comprehensive information about dentists' KAP of disaster risk management, this study will inform the development of targeted health policies aimed at improving coordinated responses to natural disasters. Such policies can facilitate training programs, enhance communication strategies, and foster interprofessional collaboration to strengthen healthcare system resilience.

## 2 Methods

### 2.1 Study Design and Settings

This cross‐sectional study was carried out at private dental offices, government and private dental clinics in Kerman city, the capital of Kerman province in southeastern Iran.

### 2.2 Study Population and Sampling

The study population included all dentists working in the aforementioned settings. A sample size of 400 dentists was determined based on Gillani et al.'s study [21], utilizing Raosoft's sample size calculation with a margin of error of 0.05, a confidence level of 95%, and a response rate of 80%. Considering a 10% dropout probability, 440 dentists were invited using the convenience sampling method.

### 2.3 Inclusion and Exclusion Criteria

The inclusion criteria required participants to be engaged in a dental practice, to hold either a general or specialized degree, and to consent to participate in this study. The exclusion criteria included incomplete questionnaires.

### 2.4 Data Collection

Data were collected using a self‐reported questionnaire administered between May 2024 and June 2024. The researchers (FKN, AMS, PJA) personally visited the study settings, distributed the questionnaires among the eligible dentists, explained the study objectives, and provided instructions on how to properly complete the questionnaires. To maximize participation, the researchers dedicated substantial time to the data collection phase and arranged a specific date for the return of the completed forms. By completing the questionnaire, participants implicitly gave their consent to participate. A total of 440 questionnaires were distributed. Of these, 421 were returned, 20 were not returned, and 17 of the returned forms were excluded due to being incomplete. In total, 403 questionnaires were included in the final analyses, yielding a response rate of 91.59%.

### 2.5 Measurement Tools

#### 2.5.1 Demographic Information Questionnaire

This questionnaire collected information about the participants' age, gender, marital status, level of education, work experience, and workplace.

#### 2.5.2 KAPs Questionnaire

The research team initially developed the KAP questionnaire using an extensive review [22, 23, 24, 25, 26, 27, 28] of the relevant literature to ensure strong content validity. The instrument was developed in Persian to ensure linguistic and cultural appropriateness for the Iranian study participants. The cut‐off values were defined by the research team for the purpose of categorizing participants' scores and facilitating data interpretation. The KAP questionnaire consisted of three domains:

1. Knowledge (14 items): Assessed using binary‐choice items (No = 0, Yes = 1). Total knowledge scores ranged from 0 to 14. Scores ranging from 0 to 5 were considered poor knowledge, scores ranging from 6 to 10 were considered moderate knowledge, and scores ranging from 11 to 14 were considered high knowledge.
2. Attitude (11 items): Assessed using a 5‐point Likert scale (1 = *strongly disagree* to 5 = *strongly agree*). Total attitude scores ranged from 11 to 55. Scores ranging from 11 to 26 indicated an undesirable attitude; scores ranging from 27 to 41 indicated a moderate attitude; and scores ranging from 42 to 55 indicated a desirable attitude.
3. Practice (7 items): Assessed using a 3‐point scale (No = 1, To some extent = 2, Yes = 3). Total practice scores ranged from 7 to 21. Scores ranging from 7 to 11 were considered poor practice, scores ranging from 12 to 16 were considered moderate practice, and scores ranging from 17 to 21 were considered desirable.

To ensure content validity [29], the questionnaire was reviewed by eight faculty members of Kerman University of Medical Sciences. Both the content validity index (CVI) and the content validity rate (CVR) were measured for each domain and the whole instrument: 0.81 and 0.83 for the knowledge questions, 0.85 and 0.80 for the attitude questions, 0.79 and 0.78 for the practice questions, and 0.82 and 0.80 for the total questionnaire. Given that all CVI values exceeded 0.78 and all CVR values exceeded 0.75, the content validity of the instrument was deemed favorable. Following the reliability assessment (internal consistency), the questionnaire was pilot‐tested on 20 dentists to assess its reliability [30]. The Cronbach's alpha coefficients were calculated for each domain and the total instrument: 0.72 for the knowledge questions, 0.81 for the attitude Questions, 0.89 for the practice questions, and 0.88 for the total questionnaire. Since all Cronbach's alpha coefficients exceeded 0.7, the questions exhibited a strong internal correlation, affirming the reliability of the questionnaire as acceptable.

### 2.6 Statistical Analysis

The demographic characteristics and the level of knowledge, attitudes, and practices of the participants were depicted via descriptive statistics, including numbers (n), percentages (%), means, and standard deviations (SD). The Kolmogorov‐Smirnov test was performed to evaluate the normality of the data, and the results confirmed a normal distribution (*p* > 0.05). Independent *t*‐test and Analysis of variance (ANOVA) were used to compare mean knowledge, attitude, practice and total scores across demographic groups. Multivariable linear regression analysis was performed to assess the effect of demographic variables on knowledge, attitude, practice, and total scores. Pearson's correlation coefficient was used to examine the relationships among knowledge, attitude, and practice. A significance level of *p* < 0.05 was considered statistically significant. Statistical analyses were conducted using the Statistical Package for the Social Sciences (SPSS) Version 26.

## 3 Results

A total of 403 dentists were included in the study. The sample was predominantly male (54.8%), with a mean age of 43.6 ± 7.9 years. The largest segment of the population fell into the 36–45 age group (43.4%). Participants reported a mean work experience of 15.3 ± 8.1 years. Other demographic characteristics are shown in Table 1.

**Table 1:** Dentists’ demographic information (*n* = 403).

Variables	Categories	*n* (%)
Gender	Male	221 (54.8)
	Female	182 (45.2)
Age	25–35 years	63 (15.6)
	36–45 years	175 (43.4)
	≥ 46 years	165 (40.9)
Marital status	Single	336 (83.4)
	Married	67 (16.6)
Level of education	General	300 (74.4)
	Specialist	103 (25.6)
Workplace	Private clinic	99 (24.6)
	Governmental clinic	71 (17.6)
	Both	233 (57.8)
Work experience	≤ 10 years	129 (32)
	11–20 years	178 (44.2)
	≥ 21 years	96 (23.8)

*Note: n* = Number, % = percentage.

**Figure 1:**
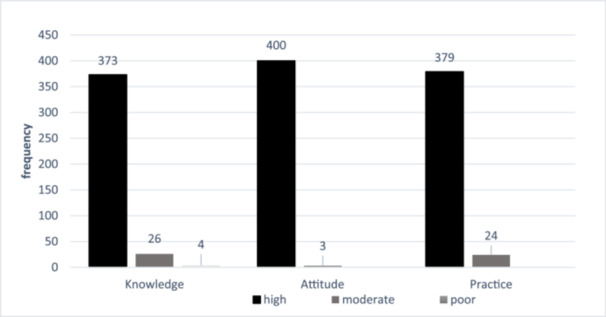
The frequency of knowledge, attitudes, and practices of dentists towards disaster risk management.

As Table 2 and Figure 1 show, dentists demonstrated high levels of knowledge (12.96 ± 1.54, 92.6%), desirable attitudes (46.42 ± 2.45, 99.3%), and desirable practices (17.63 ± 1.89, 94%) concerning natural disaster risk management.

**Table 2:** Mean scores, description, and frequency KAP in participants.

Variable	Description	*n* (%)	(Mean ± SD)
Knowledge	Poor (1–5)	4 (1)	12.96 ± 1.54
	Moderate (6–10)	26 (6.5)	
	High (11–14)	373 (92.6)	
Attitude	Moderate (27–41)	3 (0.7)	46.42 ± 2.45
	Desirable (42–55)	400 (99.3)	
Practice	Moderate (8–14)	24 (6)	17.63 ± 1.89
	Desirable (15–21)	379 (94)	

Figure 1. Frequency distribution of KAPs regarding natural disaster management among dentists in Southeastern Iran. The figure presents the number of participants categorized by their level of knowledge (poor, moderate, high), attitude (undesirable, moderate, desirable), and practice (poor, moderate, desirable) in natural disaster management. The X‐axis represents these three domains, and the Y‐axis indicates the frequency of respondents within each category. Black bars represent ‘high’ levels, dark gray bars represent ‘moderate’ levels, and light gray bars represent ‘poor’ levels.

Table 3 revealed that knowledge and attitude scores varied significantly across several demographic categories, while practice levels did not show a significant difference based on these variables. Knowledge scores were significantly different based on age group, marital status, workplace, and work experience. Individuals over 46 years of age (13.36) demonstrated the highest mean knowledge score. Married individuals (13.07) had higher knowledge scores than single individuals (12.41). Dentists employed in both private and government clinics (13.31) showed the highest knowledge score. Individuals with more than 21 years of work experience scored the highest (13.31).

**Table 3:** Dentists’ demographic information and its relationship with KAP mean scores (*n* = 403).

Variables	Categories	Knowledge (mean ± SD)	Practice (mean ± SD)	Attitudes (mean ± SD)
Gender	Male	1.62 ± 13.02	1.95 ± 17.60	2.59 ± 46.6
	Female	1.43 ± 12.90	1.82 ± 17.66	2.24 ± 46.19
	Mean difference (CI 95%)	0.12 (−0.18, 0.42)	−0.05 (−0.43, 0.31)	0.40 (−0.07, 0.88)
		*p* = 0.43	*p* = 0.75	*p* = 0.09
Age	25–35 years	1.72 ± 11.93	1.65 ± 17.34	2.2 ± 45.76
	36–45 years	1.27 ± 12.96	1.83 ± 17.62	2.46 ± 46.45
	≥ 46 years	1.55 ± 13.36	2.03 ± 17.74	2.5 ± 46.64
	η^2^ (eta squared) (CI 95%)	0.09 (0.04, 0.15)	0.01 (0.00, 0.02)	0.01 (0.00, 0.04)
		*p* < 0.001*	*p* = 0.37	*p* = 0.06
Marital status	Single	1.41 ± 12.41	1.48 ± 17.73	2.02 ± 45.89
	Married	1.54 ± 13.07	1.96 ± 17.61	2.51 ± 46.52
	Mean difference (CI 95%)	−0.65 (−1.06, −0.25)	0.11 (−0.38, 0.61)	−0.63 (−1.27, 0.01)
		*p* < 0.001*	*p* = 0.57	*p* = 0.02*
Level of education	General	1.53 ± 12.89	1.85 ± 17.57	2.52 ± 46.46
	Specialist	1.55 ± 13.17	2.01 ± 17.79	2.22 ± 46.29
	Mean difference (CI 95%)	−0.27 (−0.62, 0.06)	−0.21 (−0.64, 0.20)	0.17 (−0.37, 0.72)
		*p* = 0.11	*p* = 0.31	*p* = 0.53
Workplace	Private clinic	1.91 ± 12.53	1.86 ± 17.45	2.05 ± 45.88
	Governmental clinic	1.71 ± 12.42	1.79 ± 17.4	2.6 ± 46.21
	Both	1.18 ± 13.31	1.93 ± 17.77	2.52 ± 46.71
	η^2^ (eta squared) (CI 95%)	0.07 (0.02, 0.12)	0.01 (0.00, 0.03)	0.02 (0.01, 0.05)
		*p* < 0.001*	*p* = 0.2	*p* = 0.015*
Work experience	≤ 10 years	1.59 ± 12.31	1.7 ± 17.41	2.31 ± 45.92
	11–20 years	1.23 ± 13.25	1.95 ± 17.66	2.44 ± 46.71
	≥ 21 years	1.7 ± 13.31	2 ± 17.86	2.56 ± 46.55
	η^2^ (eta squared) (CI 95%)	0.08 (0.03, 0.13)	0.01 (0.00, 0.03)	0.02 (0.01, 0.05)
		*p* < 0.001*	*p* = 0.19	*p* = 0.013*

* Significant: Based on independent *t*‐test and ANOVA test.

The Pearson correlation coefficient revealed a significant correlation between KAPs (*p* < 0.05), as shown in Table 4. A significant, weak, and direct relationship was observed between attitude and both practice and knowledge. An increase in the attitude score was associated with an increase in both practice and knowledge scores. Similarly, a significant, moderate, and direct relationship was found between the knowledge score and the practice score, indicating that as knowledge increased, the practice score also increased.

**Table 4:** Correlation between the knowledge, attitudes, and practice scores among the dentists.

Variables	Knowledge	Attitudes	Practice
Knowledge	1	r with CI 95% = 0.12 (0.02, 0.21) *p* = 0.011	r with CI 95% = 0.41 (0.33, 0.49) *p* < 0.001
Attitudes		1	r with CI 95% = 0.15 (0.06, 0.25) *p* = 0.002
Practice			1

*Note:* *p‐values are significant at a level of ≤ 0.05.

The results of multivariate analysis revealed that employment in both private and government clinics (*β* = 0.16, *p* < 0.001) and age groups (*β* = 0.24, *p* < 0.05) contributed to a significant increase in knowledge, and working in both private and government centers (*β* = 0.13 *p* = 0.01) significantly led to a more positive attitude, shown in Table 5.

**Table 5:** Multivariate linear regression analysis for factors influencing participants’ KAP (*n* = 403).

	Knowledge		Practice		Attitude	
Variable	*β*	*p* value	*β*	*p* value	*β*	*p* value
Age	0.24	0.01	−0.001	0.98	0.06	0.48
Work experience	0.07	0.36	0.13	< 0.99	−0.004	0.96
Sex						
Male	−0.002	0.97	0.02	0.56	−0.06	0.19
Marital status						
Married	−0.05	0.35	−0.11	0.05	0.02	0.63
Workplace						
Governmental centers	0.11	0.29	0.07	0.18	0.07	0.54
Both	0.16	< 0.001	0.04	0.65	0.13	0.01
Level of education						
Specialty	0.01	0.84	0.05	0.98	−0.07	0.20
*R* ^2^	0.11		0.01		0.03	
Adjusted *R* ^2^	0.09		−0.008		0.013	

*Note: β* = standardized regression coefficients reference variable: (female, general, private clinic, single, female).

## 4 Discussion

Our study investigated Iranian dentists' KAP of natural disaster management. The results revealed high knowledge, desirable attitudes, and practices among the participants. This finding is in line with the research conducted by [7, 31], which also demonstrated a high level of KAP among their respondents regarding disaster management. These findings may be interpreted within the KAP framework, which proposes that knowledge is associated with attitude formation and practice behavior [32, 33]. Thus, the high knowledge level observed in our participants may have contributed to their favorable attitudes and better practices in disaster management. Recent evidence also supports the use of KAP in disaster‐related contexts [34, 35]. However, our findings contrast with other studies [12, 36], which reported a moderate level of KAP in disaster risk management among respondents. The high scores observed in this study suggest that dentists in Iran are relatively well‐prepared. This high objective and perceived knowledge may stem from concrete measures for engaging dental professionals in Iran's disaster management system. This level of integration is consistent with initiatives in countries such as the United States, where the role of dental professionals in disaster management is more clearly articulated. The elevated attitude scores among the respondents in this study suggest that they are willing to make significant contributions to disaster management.

The results of this study revealed that participants aged 46 years or older presented with higher levels of knowledge compared to those in other age groups. This result is in line with the studies of [12, 27, 37, 38]. It appears that seasoned dentists have encountered numerous natural emergencies and disasters throughout their careers and have learned from these experiences. In Iran, the healthcare system uses all human resources, including dentists and dental students, for disaster risk management. In the Iranian health system, dentists perform roles such as triaging victims at the incident scene and in hospitals, and identifying casualties in real mass casualty disasters and during disaster exercises; therefore, this can aid in enhancing their knowledge of natural disaster risk management.

The bivariate analysis showed that married participants had significantly higher knowledge and attitude scores than single participants, which is consistent with previous studies [39, 40]. A possible explanation is that recent disaster preparedness initiatives implemented by the Iranian health system, such as the “Household Preparedness Guide for Disasters, have placed greater emphasis on family‐based preparedness, potentially increasing awareness among married individuals. However, after adjusting for other demographic and professional characteristics in the multivariable regression model, marital status was no longer a significant independent predictor of knowledge or attitude. This suggests that the association observed in the bivariate analysis may be explained by other factors included in the model rather than by marital status itself; therefore, the relationship between marital status and disaster preparedness should be interpreted with caution.

The results of this study showed that the knowledge and attitude scores were higher among dentists working in both the private and government sectors compared to those working in only one. This finding is in line with the research of [41, 42]. This dual employment appears to foster a greater level of engagement and cross‐sector training. Dentists working in both government and private clinics likely have increased interaction with diverse colleagues and are exposed to various disaster drills and more extensive training programs across both institutional settings. This finding is supported by a survey conducted in the UAE [12], which revealed that healthcare providers possess an “average” level of knowledge about medical disasters. Their attitudes toward natural disaster risk management, however, varied based on their workplaces. Interestingly, that study found that a high level of disaster preparedness was strongly correlated with both profession and workplace.

Our study revealed that those with over 21 years of work experience presented higher knowledge scores. Researchers reported a statistically significant difference between knowledge and work experience [43, 44, 45, 46]. Furthermore, individuals with extensive work experience may possess superior knowledge and proficiency in executing routine dental procedures compared with their younger, less experienced counterparts. However, their level of knowledge about natural disaster conditions does not differ significantly from that of those with less work experience.

Our study results revealed significant correlations between KAPs. Generally, while KAPs are largely interconnected, this relationship is influenced by a variety of factors, including opinions, beliefs, personality types, demographic factors, and cultural and social influences [47]. Therefore, the discrepancies in the results of various studies can be attributed to differences in demographic variables such as geographical location, the type and severity of disasters in each region, the study year, the sample size, and the nature of the questions posed. For example, the surveys conducted in Pakistan [36] and the UAE [12] involved small dentist samples (only 14 and 17 dentists, respectively). Although KAP are closely related, their relationship may be influenced by individual beliefs, demographic characteristics, and social and cultural factors. For example, even when knowledge levels vary or are relatively low in a particular domain, practice may remain high due to strong social norms, easy access to resources, or tacit knowledge acquired through experience. This highlights the fact that the interpretation of findings should not rely solely on statistical associations but should also take into account the deeper contextual factors that influence the interaction between KAP.

### 4.1 Strengths and Limitations

One of the limitations of this research is that the study relies on self‐reported questionnaires, which introduce the risk that participants may have provided socially desirable or unrealistic answers rather than reflecting their true clinical behavior. However, by trying to explain the importance of the study objectives to the participants and considering that the survey was exclusively conducted among dentists and had a large sample size, it can be argued that any minor inaccuracies would not significantly impact the overall results. Additionally, the assessment of KAPs on a topic encompasses numerous facets and layers. Our questionnaire was only able to explore a fraction of these facets. Consequently, we made a concerted effort to cover all significant aspects of disaster risk management when designing the questionnaire. The data collection was localized to Kerman city; however, this may limit the generalizability of the findings to dentists in other regions of Iran or different international contexts.

In addition, participants were recruited using a convenience sampling method, which may have introduced selection bias. Dentists who agreed to participate may have differed from those who declined in terms of their interest in disaster preparedness or professional engagement. Consequently, the study sample may not be fully representative of all dentists, and the findings should be interpreted with caution when generalizing them to the broader dentist population.

### 4.2 Suggestions for Policy Implications

The dental profession represents a strategic capacity for integration into the national emergency response system. Accordingly, health policymakers are recommended to formally recognize the role of dentists within the national disaster management framework and to develop standardized, comprehensive protocols governing dental interventions during emergencies, with particular emphasis on vulnerable populations. Furthermore, it is imperative to reduce existing competency gaps and enhance operational readiness through the design and implementation of interdisciplinary, interactive, and simulation‐based training programs. The fulfillment of these strategic directives will significantly strengthen the resilience of the health system against both natural and man‐made hazards.

## 5 Conclusion

The high levels of knowledge, desirable practices, and positive attitudes of most Iranian dentists regarding disaster risk management indicate their significant potential to contribute to national disaster risk management. Therefore, the dental profession should be formally recognized as a vital component of Iran's emergency response infrastructure. To translate this potential into operational capacity, it is imperative to establish standardized protocols for their deployment and to integrate them into interdisciplinary disaster training programs. Capitalizing on this latent potential is imperative for fortifying the national health system's resilience and ensuring a coordinated, effective response to future disasters. To strengthen disaster preparedness among dentists, training programs and workshops should be expanded in duration and enhanced in quality, with flexible scheduling and interactive methods. Incentives for younger, less experienced dentists, along with creative approaches such as simulations or app‐based courses, are recommended. Further studies are advised to assess dentists' KAPs across various types of natural and man‐made disasters.

## Author Contributions

Fatemeh Jahanimoghadam, Hojjat Farahmandia, and Amir Mohammad Sabzi contributed to conceiving and designing the research. The data were collected, analyzed, and interpreted by Fatemeh Jahanimoghadam, Hojjat Farahmandia, Fatemeh Karami Nadik, Parya Jangipour Afshar, and Amir Mohammad Sabzi. Fatemeh Jahanimoghadam and Hojjat Farahmandia contributed equally to writing and revising the manuscript and approved the final manuscript. All authors have read and approved the final version of the manuscript. Hojjat Farahmandia had full access to all of the data in this study and takes complete responsibility for the integrity of the data and the accuracy of the data analysis.

## Funding

The authors have nothing to report.

## Ethics Statement

The present study was approved by the ethics committee of Kerman University of Medical Sciences, with the assigned code number 402000237 and the ethics code No. IR.KMU.REC. 1402.120. All steps and procedures conducted in the study adhered to the principles outlined in the Declaration of Helsinki and the guidelines set by the Committee on Publication Ethics (COPE). Necessary permissions were obtained from the study settings prior to the study.

## Consent

At the beginning of the study, all participants provided written informed consent. The participants were assured of the confidentiality of their information and voluntary participation; they could withdraw from the study at any stage without any negative consequences.

## Conflicts of Interest

The authors declare no conflicts of interest.

## Transparency Statement

The lead author, Hojjat Farahmandia, affirms that this manuscript is an honest, accurate, and transparent account of the study being reported; that no important aspects of the study have been omitted; and that any discrepancies from the study as planned (and, if relevant, registered) have been explained.

## Data Availability

The data that support the findings of this study are available on request from the corresponding author. The data are not publicly available due to privacy or ethical restrictions. The data are available upon request to the corresponding author after signing appropriate documents in line with ethical application and the decision of the Ethics Committee.
